# Relationships Among Cancer Screening Participation, E-Health Literacy, and Healthy Lifestyle Behaviors in a Group of Turkish Women: A Structural Equation Modeling Analysis

**DOI:** 10.1007/s10728-025-00525-2

**Published:** 2025-06-03

**Authors:** MİNE GÖKDUMAN KELEŞ, Eylem TOKER

**Affiliations:** 1 Burdur Mehmet Akif Ersoy University, Bucak Health High School, Deparment of Midwifery, Burdur, Türkiye; 2 Tarsus University, Faculty of Health Sciences, Department of Midwifery, Tarsus/Mersin, Türkiye

**Keywords:** E-health literacy, Healthy lifestyle behavior, Cancer screenings, Women, Structural equation modeling

## Abstract

One in five people worldwide is expected to develop cancer during their lifetime. This study aimed to examine the effect of women’s E-health literacy levels on their healthy lifestyle behaviors about their cancer screening status, within the scope of preventive cancer healthcare services in Türkiye. This cross-sectional analytical study involved 392 women, and a structural equation model (SEM) was used to identify the causal relationships between variables. Data were collected using the “E-health literacy scale” and the “healthy lifestyle behavior scale”. A structural equation model (SEM) was developed to examine the causal relationships among the variables, and the model’s fit indices and test values were analyzed and interpreted. Results showed that both E-health literacy and healthy lifestyle behavior scores were significantly higher among women who had undergone cancer screening. Additionally, women who had been screened for breast and cervical cancer demonstrated higher healthy lifestyle behavior scores. The SEM analysis indicated that E-health literacy had a positive effect on healthy lifestyle behaviors. Based on these findings, enhancing women’s E-health literacy may promote healthier lifestyle behaviors and encourage more consistent participation in preventive screenings such as cancer screenings.

## Introduction

One in five people globally will develop cancer during their lifetime, making cancer prevention a critical public health challenge of the twenty-first century. Early diagnosis can reduce cancer mortality rates by at least 40% [22]. Therefore, increasing awareness and consciousness about cancer screening in society is one of the most effective strategies for struggling with cancer. Within the context of cancer screening programs designed by the Ministry of Health in Türkiye following international guidelines, mammography is recommended every 2 years for women aged 40–69 as part of breast cancer screening. Additionally, for cervical cancer screening, women aged 30–65 are advised to have a smear and HPV-DNA test every 5 years [25]. The relevant ministry also conducts educational activities on cancer prevention and early diagnosis to ensure the target population can access these services, utilizing communication tools and media to reach individuals [25]. Therefore, this study focused on screening practices for breast and cervical cancers, which are among the most prevalent types of cancer affecting women.

Health literacy plays a vital role in promoting and protecting health. The World Health Organization (WHO) defines health literacy as the cognitive and social abilities needed to access, comprehend, and utilize health-related information, enabling individuals to enhance their health and maintain their well-being that goes beyond the fundamental skills necessary to read and understand health-related information [27]. With the advancement of technology, the concept of E-health literacy has emerged. E-health literacy is associated with the ability to handle health information from electronic resources [12, 39]. It is “the ability to search, find, understand, and evaluate health information from electronic sources and to apply the information gained to address or solve a health problem” [26]. In this context, individuals’ health literacy significantly influences women’s access to cancer-preventive health services and the development of a healthy lifestyle [1, 5–7].

The rate of internet use for accessing information is increasing globally [33]. Similarly, in Türkiye, 77.5% of women use the internet [34]. Communication technologies are extensively utilized to protect and improve individual health and provide access to health services. Furthermore, individuals’ lifestyles have a significant impact on their health [1, 14, 30]. Adopting a healthy lifestyle is crucial in cancer prevention [14].

Therefore, the level of healthy living behaviors increases with the rise in individuals’ E-health literacy [7, 10, 17, 18, 36, 37]. Cancer screenings are an essential component of healthy living behaviors. In the context of these developments, women must undergo cancer screenings and adopt healthy lifestyle behaviors such as regular exercise, a balanced diet, and avoiding smoking, alcohol, and harmful substances for the prevention or early diagnosis of cancer [13, 16].

This study examined the relationship between women’s cancer screening status, their healthy lifestyle behaviors, and E-health literacy levels and how these behaviors relate to E-health literacy. The study was guided by Pender’s Health Promotion Model—an established theoretical framework that emphasizes the multifactorial nature of adopting health-promoting behaviors. According to Pender’s model, individual characteristics, along with the ability to access and effectively use health-related information, play a critical role in promoting such behaviors [31].

Although limited evidence in the literature suggests that women who undergo cancer screening tend to exhibit higher levels of health literacy and healthier lifestyle behaviors [12, 39], no studies to date have employed structural equation modeling (SEM) to simultaneously analyze the relationship between these constructs.

These findings highlight a significant gap in the literature and underscore the need for further research exploring the relationship between E-health literacy and healthy lifestyle behaviors among women who undergo cancer screening and the relationship between these two scales through SEM.

After all these considerations, the study aims to determine the effects of E-health literacy and healthy lifestyle behaviors on women’s health screenings that will contribute to their utilization of screening program services and aid in the development and enhancement of these services, benefiting from the experiences gathered regarding these issues.

## Methods

### Objective

This study examined the effect of women’s E-health literacy and healthy lifestyle behaviors on their participation in cancer screenings and evaluated the relationship between E-health literacy and healthy lifestyle behaviors using a structural equation modeling (SEM) approach.

### Research Questions

1. Is there a significant relationship between women’s E-health literacy levels and their participation in cancer screenings?
2. Is there a significant relationship between women’s healthy lifestyle behaviors and their participation in cancer screenings?
3. Is there a significant relationship between women’s E-health literacy levels and healthy lifestyle behaviors?

### Type and Setting of the Study

This research is a descriptive, cross-sectional study. Data were collected online using Google Survey from women aged 30–65 years who voluntarily agreed to participate in the study between December 2022 and April 2023.

### Population and Sample of the Study

The target population consisted of women aged 30–65 years in Türkiye. According to data from TurkStat, 2021, there are 18,920,146 women within this age range in the country. Sample size calculation was performed using the OpenEpi program (http://www.openepi.com), which determined that a minimum of 385 participants would be required to achieve a 95% confidence interval and 80% statistical power. Ultimately, the study was completed with 392 women.

The accidental sampling method was employed. No restrictions were imposed regarding specific characteristics of the women; all women aged 30–65 (age range for cancer screening) who consented to participate were included in the study. Analysis of the sample characteristics revealed that most participants were well-educated, employed, married, and middle-aged.

*Inclusion criteria* were volunteering to participate in the study, being literate, using the internet and social media actively (WhatsApp, Instagram, Facebook, etc.), and being a woman between the ages of 30–65.

*Exclusion criteria* were refusing to participate in the study and being a woman under or over 30.

### Ethical Considerations

Ethical approval of the study was received from the Tarsus University Scientific Research and Publication Ethics Committee (decision dated 04.04.2022 and numbered 2022/26).

### Data Collection

The “Descriptive Information Form”, “E-health literacy scale”, and “healthy lifestyle behavior scale (HLBS)” were used to collect data. Women aged 30–65 were reached through the mobile network system, and data were collected online using Google Forms and were digitally stored. Participants provided informed consent via the Google Forms platform. Data collection took approximately 10–15 min for each participant.

*The Descriptive Information Form* This form was developed by researchers based on the literature [1, 8, 15, 29]. The questionnaire consisted of 13 items to collect data on participants’ socio-demographic characteristics and their awareness of CEDSTC (Cancer Early Diagnosis, Screening, and Training Centers). Cancer screening status was assessed using Question 11, which asked, “Have you ever undergone any cancer screening?” Participants who responded “yes” were directed to a follow-up question regarding the type of cancer screening they had received, while those who answered “no” were automatically directed to the E-health literacy scale section of the survey. Based on responses to this item, participants were categorized into two groups those with prior cancer screening experience and those without subsequent analyses. Descriptive results related to screening status are presented in Table 1.

**Table 1:** Participation in cancer screenings and socio-demographic characteristics of women (N = 392)

	Mean ± SD	Median (Min–Max)
Age	40.8 ± 7.3	40 (30–60)
	N	%
Education level		
Primary education	82	20.9
High school	28	7.1
University	282	71.9
Marital status		
Single	63	16.1
Married	329	83.9
Employment status		
Yes	285	72.7
No	107	27.3
Place of residence		
Village	5	1.3
District	89	22.7
City	298	76
Social insurance		
Yes	347	88.5
No	45	11.5
Income level		
Income less than expenses	30	7.7
Income equal to expenses	293	74.7
Income more than expenses	69	17.6
Daily internet usage		
1 h	147	37.5
2–3 h	236	60.2
4 h and more	9	2.3
The frequency of online searching for health-related topics		
Never	8	2
Sometimes	177	45.2
Always	207	52.8
Having cancer screening		
Yes	234	59.7
No	158	40.3
Frequency of breast cancer screenings*		
Only once	106	27
Monthly BSE	32	8.2
Periodic Breast USG	96	24.5
Frequency of cervical cancer screening*		
Only once	118	30.1
Regularly	116	29.6

*BSE:* Breast self-examination, *USG:* Ultrasonography

*n = 234 “includes only women who were screened”

*The* E-health literacy scale The Turkish version of the E-health literacy scale, originally developed by Norman and Skinner [26], was validated and tested for reliability in Turkish [33] with a Cronbach Alpha value of 0.91. It consists of two items related to internet use and eight items measuring internet attitude. The scale uses a 5-point Likert-type format (1 = strongly disagree, 5 = strongly agree). Scores range from 8 to 40, with higher scores indicating higher levels of E-health literacy [33]. The Cronbach Alpha value was calculated as 0.93.

*The Healthy Lifestyle Behavior Scale (HLBS)* The HLBS was developed by Walker and colleagues and found to have high validity and reliability by Bahar et al. [4]. The scale consists of 52 items and six subgroups: health responsibility, exercise, nutrition, interpersonal support, stress management, and self-actualization. Each item is positive and is scored on a 4-point Likert-type scale (1 = never, 2 = sometimes, 3 = often, 4 = regularly). The lowest possible score is 52, and the highest is 208, with higher scores indicating more positive, healthy lifestyle behaviors. The Cronbach alpha value of the scale in this study was 0.95.

### Data Analysis Method

To examine the effect of E-health literacy and healthy lifestyle behavior scales on women’s cancer screenings, the suitability of the data for normal distribution was first evaluated using SPSS 25.0 and the Kolmogorov–Smirnov test. For homogeneous and suitable data for normal distribution, parametric test methods (the t-test for comparisons between two different independent groups and one-way variance analysis for comparisons involving more than two variables) were used. In cases where the data did not show normal distribution, nonparametric tests (the Mann–Whitney U test was used for comparisons between two groups, and the Kruskal–Wallis test was used for comparisons involving more than two variables) were employed. Descriptive statistical data were presented using numbers, percentages, means, medians, and standard deviation values.

In examining the effect of the E-health literacy scale on the Healthy Lifestyle Behavior Total Scale and its sub-dimensions, the data were first analyzed with the SPSS 25.0 package program. This initial analysis involved establishing the appropriate questions and conducting a confirmatory factor analysis of the research model to ensure its validity. Logistic regression analysis was performed to understand the relationships between the dependent variables and the independent variables. Additionally, to explore the causal relationships between the scale variables, a structural equation model (SEM) called the “Healthy Lifestyle Model of Women’s E-health literacy” was developed using the AMOS 23 software package.

In the study, participating in cancer screening status and other sociodemographic characteristics were independent variables, while the E-health literacy and healthy lifestyle behaviors scales were considered dependent variables (for research questions 1 and 2).

**Fig. 1:**
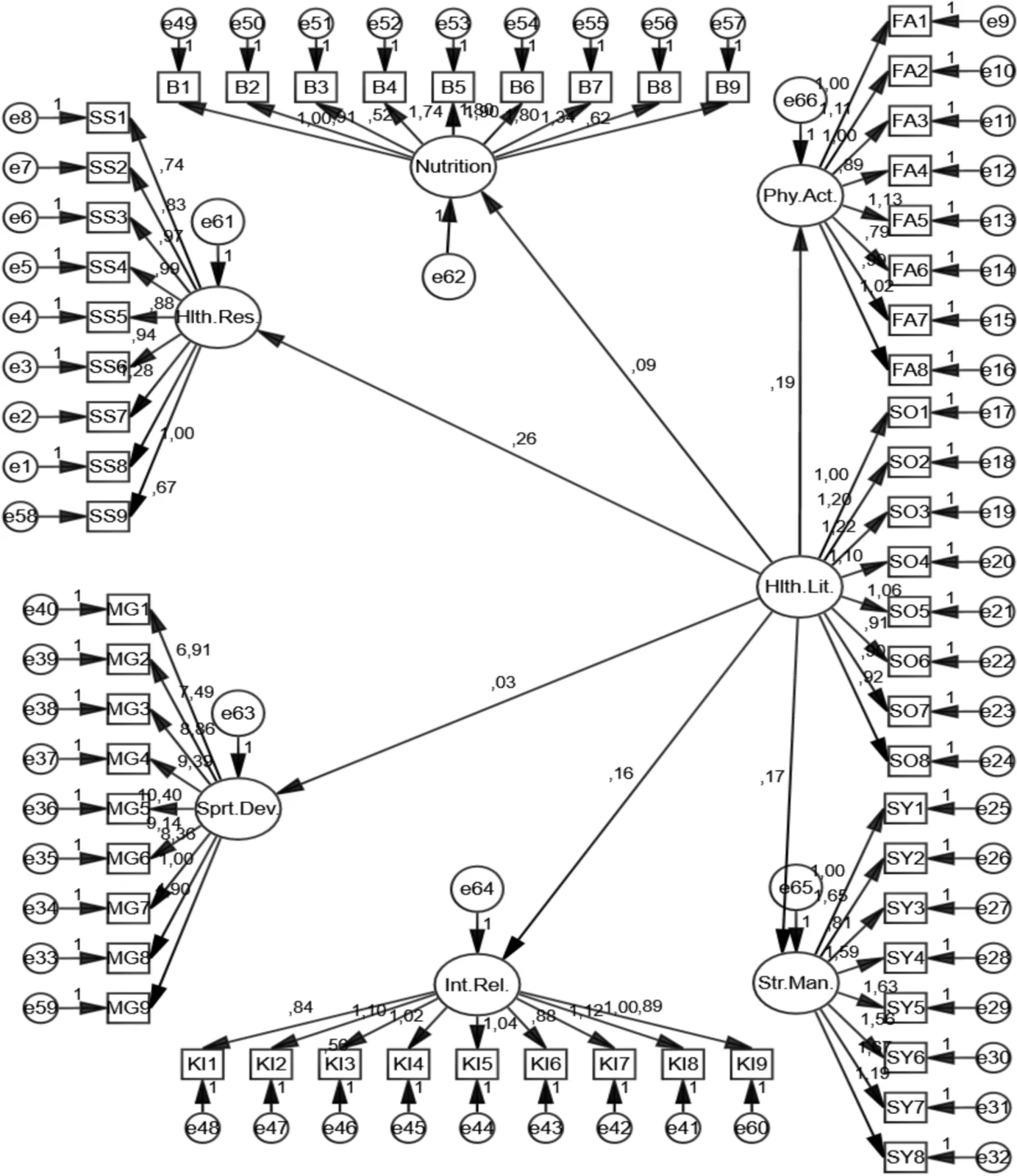
Path diagram for the effect of E-health literacy on healthy lifestyle sub-dimensions. Hilth.Lit: E-health literacy scale, Helt.Res: Health Responsibility, Sprt. Dev: Spiritual Development, Int.Rel Interpersonal Relations, Phys.Actv: Physical Activity, Str.Man: Stress Management

Additionally, structural equation modeling (SEM) was employed to evaluate the effect of E-health literacy on healthy lifestyle behaviors. The model tested direct paths from E-health literacy to each sub-dimension of healthy lifestyle behaviors. The corresponding path diagram is illustrated in Fig. 1. To further examine the influence of these variables on cancer screening participation (yes/no), E-health literacy and healthy lifestyle behavior scores were categorized and included as predictors of screening behavior. Meanwhile, within the structural equation model, E-health literacy was designated as the independent variable, and healthy lifestyle behaviors along with their sub-dimensions were specified as dependent variables (for research question 3). The fit and test values of the goodness-of-fit model were interpreted, and a significance level of *p* < 0.05 was used to interpret the data.

## Results

The study included women aged 30–65, with a mean age of 40.8 ± 7.3 years. Most participants had a university education (71.9%), were employed (72.7%), had income equal to their expenses (74.7%), and were married (72.7%). As for internet usage, 60.2% used the internet for 2–3 h daily, and only 2% never searched for health-related issues online (Table 1).

Cancer screening participation was reported by 59.7% of the participants, 8.2% had monthly check-ups, and 29.6% had regular cervical cancer screenings (Table 1).

The mean scores for the E-health literacy scale and the HLBS were moderate. Among the sub-dimensions of the HLBS, the highest score was observed in the interpersonal relationships sub-dimension, while the lowest score was seen in the stress management dimension (Table 2).

**Table 2:** The mean scores of the E-health literacy scale and Healthy Lifestyle Behaviors Scale (HLBS)

	Mean ± SD	Median (min–max)
The E-health literacy scale	30.8 ± 7.1	32 (8–40)
HLBS	156 ± 26.0	155 (75–207)
HLBS sub-dimensions		
Health responsibility	27.2 ± 5.5	27.5 (11–36)
Physical activity	19.4 ± 6.6	19 (8–32)
Nutrition	25.2 ± 5.0	25 (11–36)
Spiritual development	30.7 ± 4.6	32 (15–36)
Interpersonal relationships	30.8 ± 4.3	32 (12–36)
Stress management	22.7 ± 5.3	23 (10–32)

*HLBS:* Healthy lifestyle behaviors scale

The study examined the mean scores of the Healthy Lifestyle Behaviors Scale (HLBS) about women’s socio-demographic characteristics, internet use characteristics, and cancer screening status. It was found that women’s age, educational status, marital status, employment status, place of residence, social security, economic status, internet usage status, and the frequency of researching health-related issues on the internet did not statistically affect the mean scores of the HLBS (*p* > 0.05).

A significant difference was found between the status of having cancer screenings and the mean scores of the HLBS (*p* < 0.001). Participants who had undergone cancer screenings had higher median scores on the HLBS than those who had not been screened (Table 3).

**Table 3:** Comparison of healthy lifestyle behaviors and E-health literacy scale scores by cancer screening participation and other independent variables

	HLBS II score					E-health literacy scale				
	Mean ± SD	Median (min–max)	Test value	*p*	Dif	Mean ± SD	Median (min–max)	Test value	*p*	Dif
Variables										
1 h	159.2 ± 25.6	154 (104–207)	2.013^b^	0.365		31.8 ± 7.1	32 (8–40)	6.761^b^	0.034*	1–3
2–3 h	154.1 ± 26	155 (75–205)				30.2 ± 7.0	31 (8–40)			2–3
4 and over	154.8 ± 30.6	154 (105–197)				29.0 ± 8.6	28 (16–40)			
The frequency of online searching for health-related topics										
Never	149.5 ± 35.2	150 (103–207)	4.385^b^	0.112		21.6 ± 9.1	24.5 (8–31)	39.332^b^	0.000**	1–3
Sometimes	152.9 ± 26.1	153 (75–205)				28.9 ± 7.1	29.0 (8–40)			2–3
Always	158.9 ± 25.3	156 (105–207)				32.7 ± 6.3	34.0 (9–40)			
Cancer screening										
Yes	160.4 ± 26.2	161 (75–207)	− 4.277^a^	0.000**		31.5 ± 7.3	32 (8–40)	− 2.971^b^	0.003*	
No	149.4 ± 24.3	147.5 (89–206)				29.8 ± 6.7	30.5 (12–40)			
Frequency of breast cancer screening										
Only once (1)	153.5 ± 28.1	154 (75–207)	7.427^c^	0.001**		30.3 ± 8.1	32 (8–40)	3.841^b^	0.146	
Monthly BSE (2)	162.9 ± 18.9	163 (129–193)			1–3	32.4 ± 4.7	32 (22–40)			
Regular breast USG (3)	167.2 ± 24.4	169 (95–207)				32.5 ± 6.9	34 (8–40)			
Frequency of cervical cancer screening										
Only once	156.7 ± 25.5	154.5 (75–206)	0.320^d^	0.030*		30.9 ± 8.0	32 (8–40)	− 0.635^a^	0.525	
Regularly	164.2 ± 26.5	165.5 (95–207)				32.0 ± 6.4	32 (8–40)			

1–2: No difference (Dif.) between composites with the same number for each measured value

*HLSBS II*:Healthy lifestyle behaviors scale, *BSE* Breast self-examination

^a^Mann Whitney U

^b^Kruskal Wallis

^c^ANOVA

^d^Independent Samples T Test

*p < 0.05; **p < 0.001

There was also a significant difference in the mean scores of the HLBS based on the status of breast cancer screening (*p* < 0.001). Women who underwent intermittent breast ultrasound (USG) had higher mean scores than those who had only undergone screening once. This difference was further confirmed to be significant in detailed analysis (*p* < 0.001). Similarly, the median scores of the HLBS for women who had regular cervical cancer screenings were higher than those who had only undergone one screening, and this result was statistically significant (*p* < 0.05) (Table 3).

The average healthy lifestyle behaviors of women were compared based on their demographic characteristics, internet use, and cancer screening status. Factors such as age, marital status, education, employment, place of residence, social insurance, financial status, and breast and cervical cancer screening E-health literacy mean scores were similar across the research group, showing no statistically significant differences (*p* > 0.05).

However, the use of the internet for health-related topics significantly affected E-health literacy mean scores, with significant differences between groups. Further analysis revealed a statistically significant difference (*p* < 0.05) in mean scores between those who used the internet for four or more hours and those who used it for 1 h, 2 h, or 3 h (Table 3).

Additionally, differences were observed in E-health literacy scores among participants who used the internet for health-related issues. The advanced Dunn test showed that those who always used the internet for health-related purposes had significantly higher scores than those who never or only sometimes searched for health-related information online (*p* < 0.001) (Table 3).

According to the Structural Equation Modeling (SEM) results, E-health literacy has a significant positive effect on maintaining a healthy lifestyle (β = 0,291; *p* < 0,01).

Furthermore, E-health literacy has a significant positive effect on various specific aspects of health. According to SEM results, E-health literacy has a significant positive effect on health responsibility (β = 0.365; *p* < 0.01), physical activity (β = 0.201; *p* < 0.01), nutrition (β = 0.209; *p* < 0.05), spiritual development (β = 0.356; *p* < 0.05), interpersonal relationships (β = 0.289; *p* < 0.01) and stress management (β = 0.314; *p* < 0.01) (Table 4). The path graph of the research model derived from confirmatory factor analysis is shown in Fig. 1. It was found to be in the range of 0.66–0.93 for the E-health literacy dimension; 0.56–0.72 for the health responsibility dimension; 0.60–0.83 for the physical activity dimension; 0.17–0.65 for the nutrition dimension; 0.12–0.70 for the spiritual development dimension; 0.52–0.74 for the interpersonal relationships dimension; and 0.43–0.73 for the stress management dimension. In the analysis, no modification was performed between the error terms of the items for model improvement.

**Table 4:** Regression coefficients for the effect of the E-health literacy on healthy lifestyle sub-dimensions

Path tested			Estimate	Std. error	Critical ratio	Significance
E-health literacy	→	Healthy lifestyle	0.291	0.035	5.127	< 0.01
E-health literacy	→	Health responsibility	0.365	0.046	5.684	< 0.01
E-health literacy	→	Physical activity	0.201	0.053	3.647	< 0.01
E-health literacy	→	Nutrition	0.209	0.029	3.275	0.001
E-health literacy	→	Spiritual development	0.356	0.013	2.175	0.03
E-health literacy	→	Interpersonal relations	0.289	0.035	4.567	< 0.01
E-health literacy	→	Stress management	0.314	0.034	4.938	< 0.01

The confirmatory factor analysis goodness of fit values for the scales used in the research model are detailed in (Table 5). The analysis determined that all the scale results exhibited good fit values, indicating the robustness of the model.

**Table 5:** CFA goodness of fit values of the scales

	Χ^2^	Df	CMIN/df	*p*	RMSEA
Fit values	4115.290	1689	2.437	0.000	0.061
Acceptable	–	–	≤ 5	≤ 0.05	≤ 0.080
Good fit indices	–	–	≤ 3	≤ 0.01	≤ 0.050

## Discussion

Several studies conducted in Türkiye provide context for these findings. For instance, Gözüyeşil et al. [18] reported a total score of 142.73 ± 26.3 from the Healthy Lifestyle Scale for Women (HLSW) in a study involving women aged 15–49 years [19]. Another study by Öztoprak and Ege [28] found a total score of 119.14 ± 22.29 among female workers [28]. Similarly, Akın et al. (2022) reported a score of 129.64 ± 23.46 in a study focusing on women’s pap smear test status and healthy lifestyle behaviors [2]. These lower scores, compared to our study, are likely due to variations in the socio-demographic and cultural characteristics of the sample groups.

Additionally, research by Yılmaz et al. [38] on midwives and nurses showed that healthy lifestyle behaviors improved as the fear of breast cancer increased. Moreover, a study conducted during the pandemic by Calpbinici and Uzunkaya Öztoprak [9] reported a positive relationship between women’s attitudes towards cancer screening and healthy lifestyle behaviors. In the study, the median score of healthy lifestyle behaviors was higher for participants who underwent cancer screening (160.4 ± 26.2) than those who did not (149.4 ± 24.3). Women who had periodical breast USG had a higher median score (167.2 ± 24.4) than those who had only one screening (153.5 ± 28.1). Additionally, women who regularly had Pap smear tests for cervical cancer had significantly higher scores than those who did not. These findings suggest that individuals who engage in healthy lifestyle behaviors are also more likely to participate in cancer screenings.

Moreover, increased knowledge about cancer among women leads to higher rates of cancer screening [5, 9, 35]. Health professionals are the primary source of information on this subject, but internet use also boosts participation in cancer screening [20, 30]. In E-health literacy, individuals seek health-related information online, access and evaluate this information, and use the results to address health issues [3]. The advent of digital technologies has facilitated increased access to information on cancer screening from internet sources [3, 30]. The literature indicates that women with higher levels of health literacy are more likely to participate in cancer screenings [5, 6, 9, 32]. A study by Huang et al. in Taiwan showed a significant association between women’s health literacy and their participation in screenings for cervical, breast, and colorectal cancers [21]. Similarly, research conducted with Muslim women in Malaysia showed that higher health literacy positively influenced their attitudes toward Pap smear tests [5]. In Türkiye, studies have demonstrated that women’s awareness of gynecologic cancers increases with higher health literacy levels [6, 9]. A systematic review further established that women’s health literacy affects their behaviors regarding cancer prevention, mammography, and Pap smear testing [11]. Consistent with these findings, the current study revealed that women undergoing cancer screening also had higher E-health literacy scores. Moreover, there was a significant relationship between participants’ E-health literacy levels and both the duration and purpose of their internet use, with those using the internet for health-related information demonstrating significantly high E-health literacy, suggesting that women’s likelihood of participating in cancer screenings increases as their E-health literacy levels rise.

Structural equation modeling conducted in the study demonstrated that E-health literacy positively influences healthy lifestyle behaviors. Although the literature lacks studies that compare these scales using structural equation modeling, Liu et al. reported a positive correlation between health literacy and healthy lifestyle behaviors in patients with polycystic ovary syndrome [24]. Similarly, Keles et al. noted that digital health literacy positively impacted the quality of life after prostate cancer [23]. These findings indicate a positive correlation between women’s E-health literacy regarding health-related issues and healthy lifestyle behaviors. In this study, it was confirmed that as women’s E-health literacy increases, their engagement in healthy lifestyle behaviors also increases.

## Conclusion

The study showed that participants who underwent cancer screenings had higher healthy lifestyle behavior scores than those who did not. The likelihood of participating in cancer screenings increased as women’s E-health literacy levels improved. The highest mean E-health literacy scores were observed among those who consistently researched health-related topics online. The results indicated that women’s E-health literacy increased, so did their healthy lifestyle behaviors. Based on these findings, it is suggested that enhancing women’s E-health literacy can lead to improved healthy lifestyle behaviors and ensure regular and effective participation in essential health screenings, such as those for cancer. Therefore, it is recommended that health professionals, particularly midwives, provide education and counseling to women based on this information. Additionally, offering clear and accessible information about health protection and healthy living, especially regarding cancer screenings, on official web pages with up-to-date and evidence-based content could increase women’s participation in health screenings. To further support these efforts, healthcare professionals, including midwives, should use mobile applications and web-based platforms to provide training, information, and regular reminders to raise awareness and encourage women to participate in health screenings.

Furthermore, it is recommended that healthcare professionals, particularly midwives and nurses, take an active role in educating and counseling women about cancer prevention and screening. Interventions should emphasize the importance of accessing reliable health information, improving E-health literacy, and promoting participation in national cancer screening programs.

For future research, it is suggested that longitudinal or experimental designs be employed to assess the causal effects of E-health literacy interventions on behavior change related to cancer screening.

## Data Availability

Data from the study can be made available upon reasonable request from the corresponding author. Data from the study can be made available upon reasonable request from the corresponding author.

## Declarations

### Limitations of the Study

This study has several limitations. The findings are not generalizable beyond the study sample. Data collection was conducted via Google Forms. Additionally, variables such as cancer screening behaviors were not standardized, and the data were based on self-report.
